# High-Intensity vs Low-Intensity Knowledge Translation Interventions for Surgeons and Their Association With Process and Outcome Measures Among Patients Undergoing Rectal Cancer Surgery

**DOI:** 10.1001/jamanetworkopen.2021.17536

**Published:** 2021-07-16

**Authors:** Marko Simunovic, David Robert Urbach, Christine Fahim, Mary Ann O’Brien, Craig C. Earle, Melissa Brouwers, Evgenia Gatov, Vanja Grubac, Daniel McCormack, Nancy Baxter

**Affiliations:** 1 Department of Surgery, McMaster University, Hamilton, Ontario, Canada; 2 Department of Surgery, University of Toronto, Toronto, Ontario, Canada; 3 Li Ka Shing Knowledge Institute, Toronto, Ontario, Canada; 4 Department of Family and Community Medicine, University of Toronto, Toronto, Ontario, Canada; 5 Department of Medicine, University of Toronto, Toronto, Ontario, Canada; 6 Faculty of Medicine, School of Epidemiology and Public Health, University of Ottawa, Ottawa, Ontario, Canada; 7 ICES (formerly the Institute for Clinical Evaluative Sciences), Toronto, Ontario, Canada; 8 Institute of Health Policy, Management, and Evaluation, Dalla Lana School of Public Health, University of Toronto, Toronto, Ontario, Canada; 9 Melbourne School of Population and Global Health, University of Melbourne, Melbourne, Australia

## Abstract

**Importance:**

Surgeon-directed knowledge translation (KT) interventions for rectal cancer surgery are designed to improve patient measures, such as rates of permanent colostomy and in-hospital mortality, and to improve survival.

**Objective:**

To evaluate the association of sustained, iterative, integrated KT rectal cancer surgery interventions directed at all surgeons with process and outcome measures among patients undergoing rectal cancer surgery in a geographic region.

**Design, Setting, and Participants:**

This quality improvement study used administrative data from patients who underwent rectal cancer surgery from April 1, 2004, to March 31, 2015, in 14 health regions in Ontario, Canada. Follow-up was completed on March 31, 2020.

**Exposures:**

Surgeons in 2 regions were offered intensive KT interventions, including annual workshops, audit and feedback sessions, and, in 1 of the 2 regions, operative demonstrations, from 2006 to 2012 (high-intensity KT group). Surgeons in the remaining 12 regions did not receive these interventions (low-intensity KT group).

**Main Outcomes and Measures:**

Among patients undergoing rectal cancer surgery, proportions of preoperative pelvic magnetic resonance imaging (MRI), preoperative radiotherapy, and type of surgery were evaluated, as were in-hospital mortality and overall survival. Logistic regression models with an interaction term between group and year were used to assess whether process measures and in-hospital mortality differed between groups over time.

**Results:**

A total of 15 683 patients were included in the analysis (10 052 [64.1%] male; mean [SD] age, 65.9 [12.1] years), of whom 3762 (24.0%) were in the high-intensity group (2459 [65.4%] male; mean [SD] age, 66.4 [12.0] years) and 11 921 (76.0%) were in the low-intensity KT group (7593 [63.7%] male; mean [SD] age, 65.7 [12.1] years). A total of 1624 patients (43.2%) in the high-intensity group and 4774 (40.0%) in the low-intensity KT group underwent preoperative MRI (*P* < .001); 1321 (35.1%) and 4424 (37.1%), respectively, received preoperative radiotherapy (*P* = .03); and 967 (25.7%) and 2365 (19.8%), respectively, received permanent stoma (*P* < .001). In-hospital mortality was 1.6% (59 deaths) in the high-intensity KT group and 2.2% (258 deaths) in the low-intensity KT group (*P* = .02). Differences remained significant in multivariable models only for permanent stoma (odds ratio [OR], 1.67; 95% CI, 1.24-2.24; *P* < .001) and in-hospital mortality (OR, 0.67; 95% CI, 0.51-0.87; *P* = .003). In both groups over time, significant increases in the proportion of patients undergoing preoperative MRI (from 6.3% to 67.1%) and preoperative radiotherapy (from 16.5% to 44.7%) occurred, but there were no significant changes for permanent stoma (25.4% to 25.3% in the high-intensity group and 20.0% to 18.3% in the low-intensity group) and in-hospital mortality (0.8% to 0.8% in the high-intensity group and 2.2% to 1.8% in the low-intensity group). Time trends were similar between groups for measures that did or did not change over time. Patient overall survival was similar between groups (hazard ratio, 1.00; 95% CI, 0.90-1.11; *P* = .99).

**Conclusions and Relevance:**

In this quality improvement study, between-group differences were found in only 2 measures (permanent stoma and in-hospital mortality), but these differences were stable over time. High-intensity KT group interventions were not associated with improved patient measures and outcomes. Proper evaluation of KT or quality improvement interventions may help avoid opportunity costs associated with ineffective strategies.

## Key Points

**Question:**

Are knowledge translation (KT) interventions offered to all surgeons in a region associated with improved process and outcome measures for patients undergoing rectal cancer surgery?

**Findings:**

In this quality improvement study of 15 683 patients with rectal cancer, process and outcome measures, including use of diagnostic tests and radiotherapy and survival, were similar in the 2 groups of patients whose surgeons received high-intensity KT interventions and the 12 groups of patients whose surgeons received low-intensity KT interventions.

**Meaning:**

In this quality improvement study, high-intensity KT interventions offered to all surgeons in 2 large Ontario regions were not associated with improved measures or outcomes of rectal cancer surgery.

## Teaser

This quality improvement study uses administrative patient data from 14 regions in Ontario, Canada, to evaluate the association of sustained, iterative, integrated knowledge translation interventions for rectal cancer surgery directed at all surgeons in a geographic region with process and outcome measures among patients undergoing rectal cancer surgery.

## Introduction

Knowledge translation (KT) interventions, such as audit and feedback, are used with the expectation of improving related health care quality.^1^ However, most evaluations of KT interventions have demonstrated minimal association with targeted patient process or outcome measures.^2,3,4,5,6^ In response, stakeholders suggest that KT interventions would be more effective with the use of integrated KT approaches; theory to plan, implement, and evaluate any KT strategy; and sustained and iterative efforts.^7,8,9,10,11^ An integrated KT approach involves KT users and implementers working together to design, implement, and evaluate a study or an initiative.^7,8,9^ Key theories (eg, social learning theory) that may affect clinician behavior were used to construct the Knowledge-to-Action (KTA) Cycle.^11^ The KTA Cycle reinforces the importance of an iterative sustained effort to close quality gaps. Thus, for example, an ongoing annual audit and feedback intervention should be more effective than a single audit and feedback episode. Despite the intuitive appeal of these concepts, there has been little research at a population level that has tested associated assumptions.

Rectal cancer surgery is a useful model to test surgical quality improvement strategies because rectal cancer is a common cancer with serious clinical consequences for patients, and diagnostic, neoadjuvant treatment and surgical standards (ie, total mesorectal techniques) have changed markedly in the past 20 years.^12,13,14^ Improving the quality of rectal cancer surgery should result in higher rates of magnetic resonance imaging (MRI) scans, optimal use of preoperative radiotherapy, optimal selection of surgical procedures, and improved outcomes such as survival. Our research group has implemented population-level KT strategies to optimize rectal cancer surgery. Our most recent effort incorporated integrated KT, theory, and a sustained iterative multiyear effort.

In preparation for an evaluation of the aforementioned strategy, KT experts reviewed data on rectal cancer surgery KT interventions implemented from 2006 to 2014 in each of 14 Ontario health regions.^15^ Each region was given a score based on methods of intervention implementation (eg, integrated KT) and which interventions were implemented (eg, audit and feedback). Data were gathered using a structured interview guide that integrated an exhaustive list of KT or quality improvement interventions. Two regions had more progressive methods, a greater number of KT interventions, and high scores (mean, 78 of 100 [range, 73-83]), whereas 12 regions had low scores (mean, 30.5 of 100 [range, 22-38]). For the present study, we labeled the former and latter regions as the high-intensity and low-intensity KT groups, respectively.

The substantive changes over time in rectal cancer surgery standards and the use of high-intensity interventions in only 2 of Ontario’s 14 health regions provided an opportunity to evaluate the association of population-level KT interventions with process and outcome measures among patients undergoing rectal cancer surgery. We hypothesized that relevant measures and trends for measures would be better among patients treated by surgeons in the high-intensity group than among patients treated by surgeons in the low-intensity KT group.

## Methods

In this quality improvement study, we compared relevant measures among patients undergoing rectal cancer surgery in 2 regions of Ontario, Canada, where surgeons were offered intense rectal cancer surgery KT interventions and among those in 12 regions of Ontario where surgeons were not offered such interventions. Ethics approval for this study was obtained from the research ethics board at Sunnybrook Health Sciences Centre, Toronto, Ontario, including a waiver of informed consent because data were obtained from established administrative databases with patient, surgeon, and hospital anonymity. The study followed the Strengthening the Reporting of Observational Studies in Epidemiology (STROBE) reporting guideline.

### Study Setting and Data Sources

We included Ontario residents aged 18 to 90 years who underwent their first major resection surgery for an incident rectosigmoid or rectal cancer (*International Statistical Classification of Diseases and Related Health Problems, Tenth Revision*, with Canadian enhancements codes C19 and C20, respectively) from April 1, 2004, to March 31, 2015. We used the Ontario Cancer Registry, which enumerates every incident case of cancer in the province, for case ascertainment.^16,17^ The Canadian Institute for Health Information’s Discharge Abstract Database provided hospital-based diagnostic (*International Statistical Classification of Diseases and Related Health Problems, Tenth Revision*, with Canadian enhancements) and procedural (Canadian Classification of Health Interventions) information.^18^ The Cancer Activity Level Reporting registries were used to identify cancer-related treatments. We obtained demographic information from the Registered Person’s Database. Data sets were linked at the individual level using unique encoded identifiers and analyzed at ICES (formerly known as the Institute for Clinical Evaluative Sciences).

### Study Groups and Patient Population

In a previous study,^15^ KT experts demarcated Ontario’s 14 health regions into 2 groups based on the scoring of surgeon-directed KT methods and interventions designed to improve the quality of rectal cancer surgery that were potentially used from 2006 to 2014. In 2 regions, academic surgeons offered sustained, iterative, integrated KT interventions to all front-line surgeons in their respective regions from 2006 to 2012.^19,20^ Surgeons and patients in these 2 regions constituted the exposed group and were labeled the *high-intensity KT group*. Front-line surgeons helped organize selection of measures for audit and feedback and planning of meetings (ie, integrated KT methods). Interventions included audit and feedback of relevant measures, with feedback occurring at annual workshops (ie, KTA-Cycle–like approaches). In 1 high-intensity region, surgeons were also encouraged to invite surgeon researchers to assist with surgery at the home hospital of the participating surgeon (ie, an operative demonstration). In both regions, an ongoing explicit effort was made to improve patient outcomes at the region level. Engagement with any intervention by front-line surgeons was voluntary. Most efforts focused on expert surgery (good-quality mesorectal excision to possibly lower rates of permanent stoma and local tumor recurrence), greater use of preoperative pelvic imaging with MRI, and encouraging, when appropriate, the use of preoperative vs postoperative radiotherapy. At any time in the 2 high-intensity regions, approximately 60 surgeons provided rectal cancer surgical procedures. Surgeons and patients in the 12 remaining regions constituted the unexposed group and were labeled the *low-intensity KT group*. In these regions, no ongoing region-level, surgeon-directed KT rectal cancer surgery interventions occurred.

Patients were attached to the high-intensity or low-intensity region where their rectal cancer surgery occurred. The start date of April 1, 2004, ensured capture of cases before relevant activities in the high-intensity KT group. We excluded individuals who underwent a resection more than 2 months before or more than 8 months after the date of their diagnosis. Captured cases outside these 2 intervals likely reflected surgery with an unknown cancer diagnosis or surgery for palliative reasons, respectively; both scenarios could influence process and outcome measures. Other exclusion criteria included a hospital stay of less than 2 days, which would be too short for patient surgery and recovery, and procedures performed in facilities in which 3 or fewer resections for rectal cancer were performed annually for patient confidentiality reasons.

### Patient and Hospital Variables

We determined patient age (grouped as ≤50, 51-65, 66-80, and ≥81 years), sex, area-based income quintile, and urban or rural dwelling. Area-based income was assessed at the level of dissemination areas based on individual postal code. We determined whether patients’ surgery was elective or urgent and whether it resulted in an anastomosis (eg, low anterior resection), potentially reversible stoma (eg, low anterior resection and diverting ileostomy or Hartman resection), or permanent stoma (eg, abdominoperineal resection). We also captured TNM cancer stage at the time of surgery (data comprehensively available after 2007 only) and medical comorbidities present on the index admission using the Charlson Comorbidity Index.^21^ In addition, we identified hospital teaching status (yes or no) and hospital procedure volume, grouped into low-, low-medium–, medium-high–, or high-volume institutions. All Ontario hospitals were first ranked and then placed into volume categories, with each group containing approximately equal numbers of patients.

### Process and Outcome Measures

Process measures of interest were pelvic MRI 5 months or less before surgery, preoperative radiotherapy administered 5 months or less before surgery, postoperative radiotherapy use 6 months or less after surgery, and type of surgery (resection with anastomosis, resection with reversible stoma, and resection with permanent stoma). Time intervals ensured processes were associated with incident cases of rectal cancer surgery. Preoperative pelvic MRI is accepted as the best test to determine whether a patient has a stage II or III tumor and is thus a candidate for preoperative radiotherapy.^13^ The use of radiotherapy lowers the risk of local tumor recurrence but also confers a risk of serious adverse effects, including bowel and sexual dysfunction.^22,23^ Because the benefits and risks of radiotherapy must be balanced, in the absence of a criterion standard rate, a priori we did not know whether superior use of radiotherapy at a group level would be associated with a higher or lower proportion of patients receiving radiotherapy. Of note, preoperative radiotherapy is associated with lower rates of local recurrence than postoperative radiotherapy.^24^ Most patients would prefer to avoid a permanent stoma and instead receive an anastomosis (ie, reconnection of the bowel).^13^ A guideline^13^ recommends that patients with a low anastomosis receive a potentially reversible stoma (eg, ileostomy) to decrease the long-term risk of a permanent stoma despite closure of the ileostomy requiring an additional surgery. Outcomes of interest were rates of in-hospital mortality and overall survival among patients discharged from the hospital.

### Statistical Analysis

We used 2-tailed *t* and χ^2^ tests for between-group comparisons of patient and hospital characteristics, process measures, and in-hospital mortality. We used logistic regression models with an interaction term between group and year to determine whether time trends process measures and in-hospital mortality differed between groups. We used multivariable hierarchical logistic regression models to examine the association of study group with measures after adjustment for patient age, sex, income, rural dwelling, admission category, the presence of comorbidities, cancer type (rectosigmoid or rectal), hospital volume group, hospital teaching status, and year of surgery. We used generalized estimating equations with robust SEs to account for clustering of patients within hospitals in all models. To examine the association of group with survival, we used a multilevel Cox proportional hazards regression model adjusting for the aforementioned covariates. This model used a frailty approach to account for patient clustering within hospitals. Patients were followed up until death or March 31, 2020. We used adjusted hierarchical logistic regression models to determine potential differences between groups over time in slopes. As a sensitivity analysis, we examined the effects of adding cancer stage as a covariate to all logistic and Cox proportional hazards regression models for patients diagnosed after 2007, when staging data became available. Statistical analyses were conducted using SAS statistical software, version 9.4 (SAS Institute Inc) from January 1, 2019, to December 31, 2020. Two-sided *P* < .05 indicates statistical significance.

## Results

**Figure 1.**
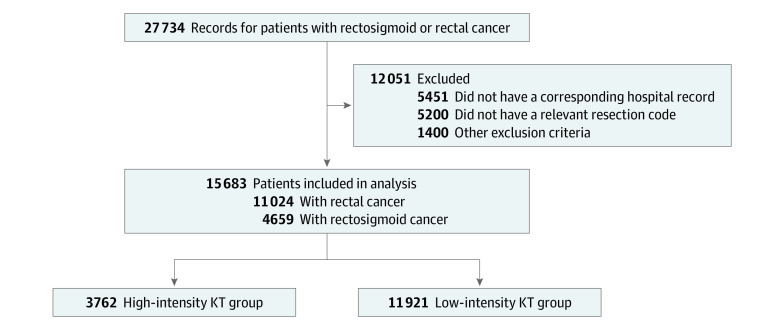
Study Flowchart KT indicates knowledge translation.

A total of 27 734 records for patients with incident rectosigmoid or rectal cancer diagnosed from April 1, 2004, to March 31, 2015, were captured in the Ontario Cancer Registry. We excluded 12 051 patients owing to 5451 not having a corresponding hospital record, 5200 not having a relevant resection code, and 1400 meeting the remaining exclusion criteria (Figure 1). The final study cohort thus included 15 683 patients (mean [SD] age, 65.9 [12.1] years; 5631 [35.9%] female and 10 052 [64.1%] male). Among these patients, 4659 (29.7%) had a diagnosis of rectosigmoid and 11 024 (70.3%) had a diagnosis of rectal cancer. The high-intensity KT group included 3762 patients (24.0%; 2459 [65.4%] male; mean [SD] age, 66.4 [12.0]), and the low-intensity KT group included 11 921 patients (76.0%; 7593 [63.7%] male; mean [SD] age, 65.7 [12.1] years). Between groups, there were modest differences in some variables, including patient age category and Charlson Comorbidity Index score, but not in others, such as admission status, tumor type, and, when available, tumor stage (Table 1). Rates of high-volume hospital cases (2039 [54.2%] vs 4383 [36.8%]; *P* < .001) and teaching hospital cases (2467 [65.6%] vs 3733 [31.3%]; *P* < .001) were higher in the high-intensity KT group than in the low-intensity KT group.

**Table 1.** Characteristics of Patients, Tumors, and Hospitals by KT Intensity Group^a^

Characteristic	Patients		
	High-intensity KT group (n = 3762)	Low-intensity KT group (n = 11 921)	All (N = 15 683)
**Patient characteristics**			
Age category, y			
≤50	365 (9.7)	1352 (11.3)	1717 (10.9)
51-65	1324 (35.2)	4311 (36.2)	5635 (35.9)
66-80	1598 (42.5)	4886 (41.0)	6484 (41.3)
≥81	475 (12.6)	1372 (11.5)	1847 (11.8)
Sex			
Female	1303 (34.6)	4328 (36.3)	5631 (35.9)
Male	2459 (65.4)	7593 (63.7)	10 052 (64.1)
Income quintile^b^			
1 (lowest)	636 (16.9)	2243 (18.8)	2879 (18.4)
2	858 (22.8)	2450 (20.6)	3308 (21.1)
3	724 (19.2)	2302 (19.3)	3026 (19.3)
4	797 (21.2)	2507 (21.0)	3304 (21.1)
5 (highest)	734 (19.5)	2386 (20.0)	3120 (19.9)
Urban (vs rural)^b^	3325 (88.4)	10 085 (84.6)	13 410 (85.5)
Elective admission (vs urgent)	3441 (91.5)	10 884 (91.3)	14 325 (91.3)
Charlson Comorbidity Index score, mean (SD)	0.39 (0.83)	0.34 (0.76)	0.35 (0.78)
**Tumor characteristics**			
Cancer type			
Rectosigmoid	1120 (29.8)	3539 (29.7)	4659 (29.7)
Rectal	2642 (70.2)	8382 (70.3)	11 024 (70.3)
Cancer stage^c^			
I	673 (17.9)	1996 (16.7)	2669 (17.0)
II	684 (18.2)	2298 (19.3)	2982 (19.0)
III	1175 (31.2)	3633 (30.5)	4808 (30.7)
IV	217 (5.8)	744 (6.2)	961 (6.1)
Missing	1013 (26.9)	3250 (27.3)	4263 (27.2)
**Hospital characteristics**			
Procedure volume			
Low	1017 (27.0)	2971 (24.9)	3988 (25.4)
Low-medium	318 (8.5)	2106 (17.7)	2424 (15.5)
High-medium	388 (10.3)	2461 (20.6)	2849 (18.2)
High	2039 (54.2)	4383 (36.8)	6422 (40.9)
Teaching status			
Community	1295 (34.4)	8188 (68.7)	9483 (60.5)
Teaching	2467 (65.6)	3733 (31.3)	6200 (39.5)

Abbreviation: KT, knowledge translation.

^a^ Data are from patients who underwent major rectosigmoid or rectal cancer surgery from April 1, 2004, to March 31, 2015. Unless otherwise indicated, data are expressed as number (percentage) of patients.

^b^ Missing less than 0.5%.

^c^ Data not available before 2007.

Preoperative MRI was performed for 1624 patients (43.2%) in the high-intensity KT group and 4774 (40.0%) in the low-intensity KT group (*P* < .001); preoperative radiotherapy was administered to 1321 (35.1%) and 4424 (37.1%), respectively (*P* = .03); postoperative radiotherapy was administered to 497 (13.2%) and 1917 (16.1%), respectively (*P* < .001); reversible stoma was performed for 1366 (36.3%) and 4472 (37.5%), respectively (*P* = .18); and permanent stoma was performed for 967 (25.7%) and 2365 (19.8%), respectively (*P* < .001). In-hospital mortality was 1.6% (59 deaths) in the high-intensity KT group and 2.2% (258 deaths) in the low-intensity KT group (*P* = .02) (Table 2). In multivariable models, differences in measures were not significant with 2 exceptions (Table 2). In the high-intensity KT group, the odds were greater for permanent stoma (odds ratio [OR], 1.67 [95% CI, 1.24-2.24]; *P* < .001) and lower for in-hospital mortality (OR, 0.67 [95% CI, 0.51-0.87]; *P* = .003). Patient overall survival was similar in the high-intensity and low-intensity KT groups (hazard ratio, 1.00 [95% CI, 0.90-1.11]; *P* = .99). Sensitivity analyses demonstrated that additional adjustment for cancer stage did not affect the significance, magnitude, or direction of the association for any process measure, in-hospital mortality, or overall survival.

**Table 2.** Process and Outcome Measures of Rectosigmoid or Rectal Cancer Resection by KT Intensity Group^a^

Measures	KT group, No. (%)		*P* value^b^	Adjusted analysis^c^	
	High intensity (n = 3762)	Low intensity (n = 11 921)		OR (95% CI)	*P* value^b^
Preoperative MRI	1624 (43.2)	4774 (40.0)	<.001	1.27 (0.62-2.60)	.51
Radiotherapy					
Preoperative	1321 (35.1)	4424 (37.1)	.03	0.76 (0.58-0.99)	.05
Postoperative	497 (13.2)	1917 (16.1)	<.001	0.87 (0.68-1.15)	.27
Stoma					
Potentially reversible	1366 (36.3)	4472 (37.5)	.18	0.79 (0.60-1.04)	.10
Permanent	967 (25.7)	2365 (19.8)	<.001	1.67 (1.24-2.24)	<.001
In-hospital mortality	59 (1.6)	258 (2.2)	.02	0.67 (0.51-0.87)	.003

Abbreviations: KT, knowledge translation; MRI, magnetic resonance imaging; OR, odds ratio.

^a^ Data are from patients who underwent major rectosigmoid or rectal cancer surgery from April 1, 2004, to March 31, 2015.

^b^ Odds ratios for the high-intensity KT group vs the low-intensity KT group were obtained using hierarchical logistic regression models (patients within hospitals) adjusted for age, sex, income, rural dwelling, admission category, the presence of comorbidities, cancer type, year of surgery, and hospital teaching status and volume.

^c^ Calculated using the χ^2^ test.

**Figure 2.**
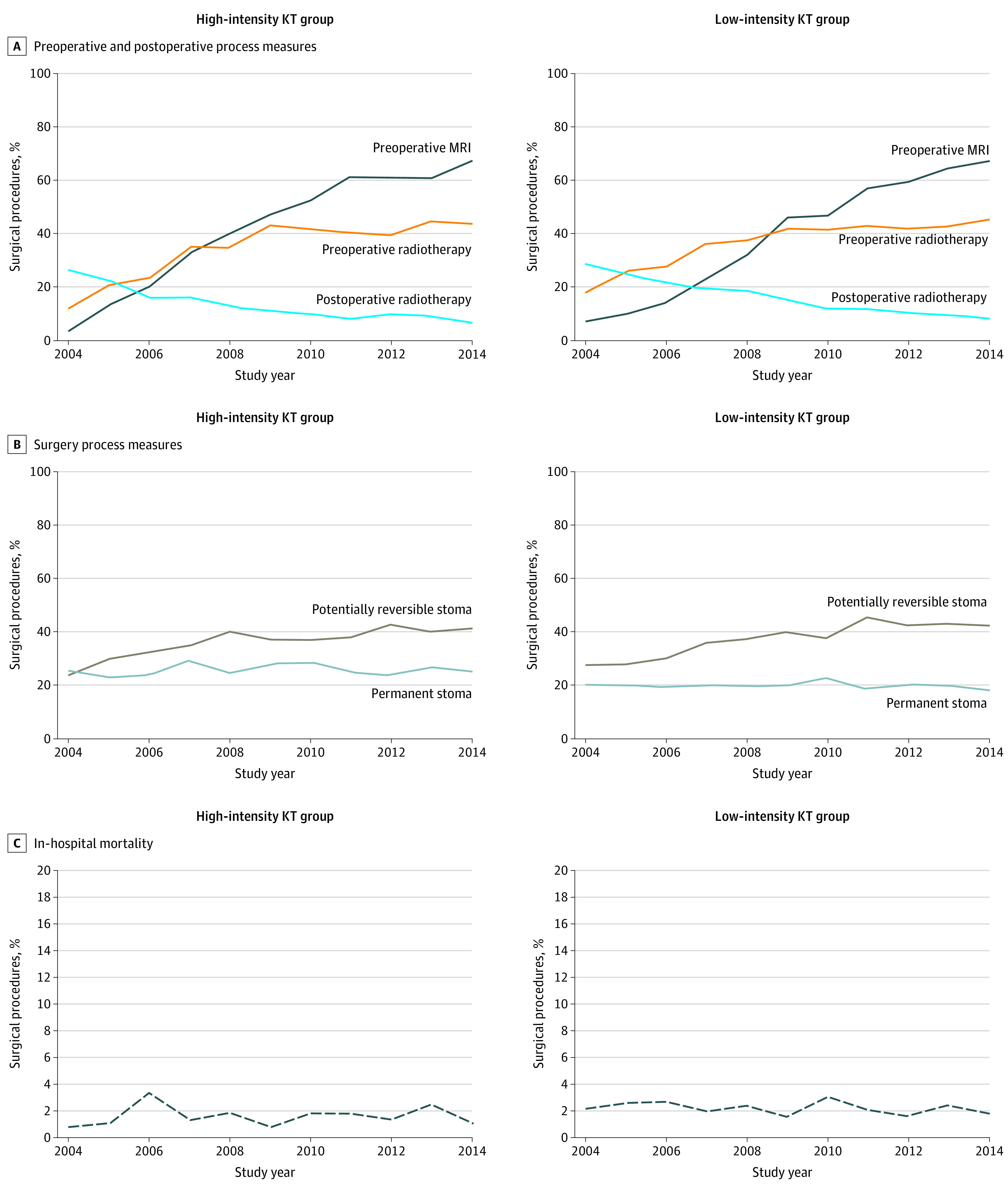
Process and Outcome Measures of Rectosigmoid or Rectal Cancer Surgery by Knowledge Translation (KT) Intensity Group Data were accrued from April 1, 2004, to March 31, 2015. In logistic regression models, *P* values were not significant for the interaction term KT group × year for all measures, indicating no differences in time trends between groups. Slopes were obtained from fully adjusted hierarchical logistic regression models. A and B, *P* < .05 for slope. For time trend differences between groups, *P* = .54 for preoperative magnetic resonance imaging (MRI); *P* = .17 for preoperative radiotherapy; *P* = .99 for postoperative radiotherapy; *P* = .46 for permanent stoma; and *P* = .68 for potentially reversible stoma. C, *P* = .64 for slope; *P* = .43 for differences between groups over time.

Over time, marked increases occurred in the use of MRI, preoperative radiotherapy, and temporary stoma (Figure 2). For example, the proportion of preoperative MRI increased from approximately 6.3% to 67.1%, the proportion of preoperative radiotherapy increased from 16.5% to 44.7%, and the proportion of temporary stoma increased from 26.9% to 42.0%. In contrast, the proportion of permanent stoma (25.4% to 25.3% in the high-intensity group and 20.0% to 18.3% in the low-intensity group) and in-hospital mortality (0.8% to 0.8% in the high-intensity group and 2.2% to 1.8% in the low-intensity group) remained similar (Figure 2). There were no significant time trend differences between groups for measures that did or did not change over time. In multivariable models controlling for group, changes in slope were significant for MRI (0.33; *P* < .001), preoperative radiotherapy (0.11; *P* < .001), and temporary stoma (0.08; *P* < .001) and were not significant for permanent stoma (0.0002; *P* = .98) and in-hospital mortality (−0.01; *P* = .64).

## Discussion

In this quality improvement study, we found that the resource-intense methods and interventions used in the high-intensity KT regions were not associated with improved patient measures. In multivariable models, there were no significant differences between groups in most measures, including survival. There were marked changes in proportions over time for use of MRI, preoperative radiotherapy, and temporary stoma. However, changes occurred in parallel for patients in the high-intensity and low-intensity KT groups. There were modest differences in proportions between groups for permanent stoma and in-hospital mortality. However, absolute values and between-group differences for these 2 measures did not change significantly over time. The KT methods and interventions used in the high-intensity KT regions were not associated with significant improvements in patient process or outcome measures regardless of whether such measures changed (eg, use of MRI) or did not change (eg, in-hospital mortality) over time.

These results are consistent with those of other KT studies^25,26^ including a small number of KT strategies that used intense, sustained integrated KT methods to improve management of chronic diseases. The findings suggest a paradox in quality improvement: although marked changes in process measures and patient outcomes over time can occur, the mechanisms or factors associated with such changes are not fully understood; thus, how to initiate or accelerate desired changes is unknown. This paradox indicates the importance of rigorous ongoing evaluation of any KT or quality improvement intervention designed to influence care, especially at the population level.^27^ The methods and interventions used in the high-intensity KT regions were not associated with improved study measures. Other approaches are likely needed to close quality gaps (eg, to lower rates of permanent stoma). Such approaches may include modifications of the KT strategies used in the high-intensity KT regions or approaches not yet defined. However, any efforts should be evaluated properly. All interventions are associated with financial and opportunity costs. A lack of proper transparent evaluation may result in ongoing resource use for ineffective interventions.

Quality improvement activities led by Cancer Care Ontario (CCO), the governing body responsible for the quality of cancer care across Ontario, may have been associated with changes in some of the study measures. During the years of our study, CCO organized numerous interventions to improve rectal cancer surgical care across Ontario that can be described as KT interventions, including guidelines, audit and feedback of quality markers, encouragement of multidisciplinary cancer conferences, and communities of practice interventions.^28,29,30,31^ However, experts concluded that there were minimal rectal cancer surgery KT interventions in 12 of Ontario’s 14 health regions during the years reviewed.^15^ In addition, the dates of release of CCO guidelines relevant to rectal cancer surgery do not correspond with the changes observed in our study. For example, a guideline on the use of preoperative pelvic MRI was published in 2016,^32^ and a guideline on preoperative vs postoperative radiotherapy was published in 2008,^33^ which is when increased use of preoperative radiotherapy leveled in our study. Also, no CCO guideline to date has addressed use of temporary stoma.

### Limitations

This study has limitations. First, this was a quality improvement study using administrative data, which may not comprehensively capture relevant variables and measures or important case mix differences between groups. However, the databases used in this study are accurate for key data points, such as patient characteristics, procedures provided, and mortality status, limiting potential confounding.^17,34,35^ Our main results are also based on multivariable models. In addition, previous research^36^ showed that patients with rectal cancer were frequently treated in their health region of origin. Patient and tumor characteristics are unlikely to vary markedly among regions, and this was supported by our results.^36^ Second, full tumor staging for all patients was available only after 2007. However, sensitivity analyses with inclusion of this variable for patients treated after 2007 did not change the magnitude or direction of results. Third, important rectal cancer surgery measures, such as local tumor recurrence, were not evaluated in this study. Obtainment of this measure requires direct review of medical records, which was beyond the scope of the present study. Fourth, we cannot be certain that the KT methods and interventions used in the high-intensity KT group were ideally implemented. Although our results are consistent with other resource-intense KT intervention studies, a method of measuring implementation fidelity would have been helpful.^37,38,39^ Fifth, the KT methods and interventions used in this study or variations of such strategies may have different effects in other jurisdictions or other clinical areas. We encourage more KT strategy research including appropriate concurrent evaluation.

## Conclusions

In this quality improvement study, high-intensity KT methods and interventions offered to all surgeons in 2 of 14 health regions in Ontario from 2006 to 2012 were not associated with improvements in patient process or outcome measures. Proper evaluation of KT or quality improvement interventions may help avoid opportunity costs associated with ineffective strategies.
